# Prevalence and Relevance of Vitamin D Deficiency in Newly Diagnosed Breast Cancer Patients: A Pilot Study

**DOI:** 10.3390/nu15061450

**Published:** 2023-03-17

**Authors:** Cosima Zemlin, Laura Altmayer, Caroline Stuhlert, Julia Theresa Schleicher, Carolin Wörmann, Marina Lang, Laura-Sophie Scherer, Ida Clara Thul, Lisanne Sophie Spenner, Jana Alisa Simon, Alina Wind, Elisabeth Kaiser, Regine Weber, Sybelle Goedicke-Fritz, Gudrun Wagenpfeil, Michael Zemlin, Erich-Franz Solomayer, Jörg Reichrath, Carolin Müller

**Affiliations:** 1 Department of Gynecology, Obstetrics & Reproductive Medicine, Saarland University Medical Center, 66421 Homburg, Germany; 2 Department of General Pediatrics and Neonatology, Saarland University Medical Center, 66421 Homburg, Germany; 3 Institute for Medical Biometry, Epidemiology and Medical Informatics (IMBEI), Saarland University, Campus Homburg, 66421 Homburg, Germany; 4 Department of Dermatology, Venereology and Allergology, Saarland University Medical Center, 66421 Homburg, Germany

**Keywords:** vitamin D, prognostic factors, breast cancer, nutrition

## Abstract

(1) Background: Vitamin D plays an important role in many types of cancer. It was the aim of this study to analyze serum 25-hydroxyvitamin D (25(OH)D) levels in newly diagnosed breast cancer patients, and the association with prognostic and lifestyle factors. (2) Methods: 110 non-metastatic breast cancer patients were included in the prospective observational “BEGYN” study at Saarland University Medical Center between September 2019 and January 2021. At the initiation visit, serum 25(OH)D levels were measured. Clinicopathological data on prognosis, nutrition, and lifestyle were extracted from data files and obtained using a questionnaire. (3) Results: Median serum 25(OH)D in breast cancer patients was 24 ng/mL (range 5–65 ng/mL), with 64.8% of patients being vitamin D deficient. 25(OH)D was higher among patients that reported the use of vitamin D supplements (43 ng/mL versus 22 ng/mL; *p* < 0.001), and in summer compared to other seasons (*p* = 0.03). Patients with moderate vitamin D deficiency were less likely to have triple negative breast cancer (*p* = 0.047). (4) Conclusions: Routinely measured vitamin D deficiency is common in breast cancer patients and needs to be detected and treated. However, our results do not support the hypothesis that vitamin D deficiency may be a main prognostic factor for breast cancer.

## 1. Introduction

The benefits of nutritional supplements, immune stabilizing micronutrients or vitamins in cancer patients remain controversial [1]. Many patients believe that the intake of micronutrients or vitamins could improve their health status, and the intake of nutritional supplements by oncological patients has continued to increase in recent years [1]. Depending on the study population, 30–90% of oncological patients take supplements or presumably immunoprotective micronutrients [1,2], usually without the knowledge of their physician [1]. One vitamin that has become increasingly popular in recent years is vitamin D.

As patients’ vitamin levels are not determined in clinical routine, many patients may unknowingly suffer from vitamin deficiency. Vitamin D deficiency can be subgrouped into mild, moderate, or severe vitamin D deficiency (serum 25-hydroxyvitamin D 25(OH)D < 30 ng/mL, <20 ng/mL, and <10 ng/mL, respectively) [3]. In particular, moderate and severe vitamin D deficiency are associated with unfavorable skeletal outcomes (e.g., fractures or bone loss) and increased mortality [3]. In an analysis of vitamin D status in 2267 German women, nearly two thirds had a severe, moderate, or mild vitamin D deficiency [4]. Early treatment of vitamin D deficiency is important to prevent long-term complications such as bone loss. In addition, vitamin D deficiency is associated with cardiovascular diseases, metabolic syndrome, impaired cognitive function, and depression [5,6].

Vitamin D and its possible role in cancer development has been very controversial in recent years [7]. The important role of vitamin D in cell differentiation, as well as anti-inflammatory effects and antiproliferative effects, is already known [8]. Animal and preclinical studies prove the presence of vitamin D receptors in breast cancer cells and see an association between vitamin D levels and breast cancer development [9,10]. Among other things, one assumed effect of vitamin D on the Th17 lymphocytes is to influence tumor growth and metastases in murine models [11]. Moreover, the combination of calcitriol with antineoplastic drugs showed beneficial effects in preclinical studies [12]. However, results of clinical trials in patients are inconsistent and the “true” role of vitamin D in breast cancer evolution is still not known [8,9].

The aim of this study was to determine serum 25-hydroxyvitamin D (25(OH)D) levels of newly diagnosed breast cancer patients. Furthermore, as some studies link especially aggressive breast cancer to low serum 25-hydroxyvitamin D (25(OH)D) levels [13,14], we correlated 25(OH)D levels with prognostic factors as well as potential modulators (e.g., supplementation, sun exposure). Several prognostic factors associated with a poor outcome in breast cancer patients have already been identified, including high grading and a high value of the proliferation marker Ki67. In addition, locally advanced breast cancer with bigger tumor size (cT3/4) or positive lymph nodes (pN+) is related to poor outcome and a high risk of locoregional or metastatic relapses [15,16,17,18,19,20].

Breast cancer can be divided into four different subgroups dependent on immunohistochemistry: hormone receptor (estrogen/progesterone) positive, human epidermal growth factor-2 (Her2) negative breast cancer with low Ki67 < 25 (Luminal A) and high Ki67 ≥ 25 (Luminal B); Her2 positive tumors; and triple negative (estrogen, progesterone and Her2 negative) breast cancer [21]. The different breast cancer subtypes have an impact on patients’ outcome [16]. In particular, patients with aggressive tumor biology, like triple negative breast cancer, have worse outcomes and shorter overall survival [22].

## 2. Materials and Methods

### 2.1. Data Collection

The BEGYN study was approved by the Ethics committee of the Medical Association of Saarland (study # 229/18). This prospective observational study included 110 non-metastatic breast cancer patients recruited between September 2019 and January 2021 at Saarland University Medical Center. Inclusion criteria were age > 18 years, sufficient knowledge of German language (to fill out questionnaires), and a physical condition that allows spirometry on the treadmill. The ability to use a smartphone and fitness tracker was required as well. All patients gave a written declaration of consent to participate in the study. Pregnant patients, patients suffering from metastases or simultaneous carcinomas (in other locations), and patients with a life expectancy of less than one year were excluded.

At the initiation visit (before the start of any pharmacologic or operative therapy), serum concentration of 25(OH)D was measured with using the LIAISON^®^ 25 OH Vitamin D TOTAL Assay (DiaSorin, 13040 Saluggia, Italy, REF 310,600). Supplementation of vitamins and trace elements was documented, and patients received a questionnaire about sun exposure and nutrition. Data were collected as described previously in the BEGYN study protocol [23]. Clinical characteristics (e.g., age, BMI, Karnofsky performance status scale) and histopathological parameters (e.g., TNM classification using the UICC 8th Edition [24], Grading: Elston and Ellis 1991 [25], Immunohistochemistry for determination of estrogen receptor (ER), progesterone receptor (PR), Her2 and Ki67 [21,26]) were documented.

### 2.2. Statistics

Statistical analyses were performed using SPSS 28.0 (IBM, Armonk, NY, USA). Qualitative parameters (e.g., tumor stage) are presented as frequencies. Quantitative parameters are given as mean with standard deviation or as median with range. The Kolmogorov–Smirnov test was used to test for normal distribution. Univariate linear regression was performed to analyze possible modulators of 25(OH)D levels. To determine whether various prognostic factors in breast cancer are related to 25(OH)D levels, the patients were divided into 3 groups: patients with at least mild (≤/>30 ng/mL), at least moderate (≤/>20 ng/mL), or severe (≤/>10 ng/mL) vitamin D deficiency. A possible association between vitamin D levels and the prognostic factors was examined using the Mann–Whitney U test for metric and ordinal variables with asymptotic p-value. For the group with severe vitamin D deficiency, exact p-value was used due to small sample size. Nominal variables were tested with crosstabulation and the Chi-Square test as well as Fishers’ exact *p*-value.

## 3. Results

A total of 110 non-metastatic breast cancer patients participated in the BEGYN study between September 2019 and January 2021 [23]. The median patients’ age was 54 years (range 26–81). Five patients (4.5%) suffered from bilateral breast cancer. Whereas 94 patients (85.5%) had their first malignant disease, 16 patients (14.5%) suffered from recurrent cancer or a second carcinoma. The patients were in good general condition: 102 patients (92.7%) indicated a Karnofsky performance status scale of 90 or 100% and thus had no or minimal limitations. Eight patients (7.3%) had a Karnofsky performance status scale of 80% (they could engage in normal activity with effort). The median BMI was 26 (range 19–39). A total of 42 patients (38.2%) were previous smokers or still smoking, with a median of 17 pack years (range 1–58). Moderate alcohol consumption was reported by 101 patients (91.6%). Tumor biology, tumor entity, and tumor stage are given in Table 1, Table 2 and Table 3. Ki67 index was 29 (range 1–90).

**Figure 1:**
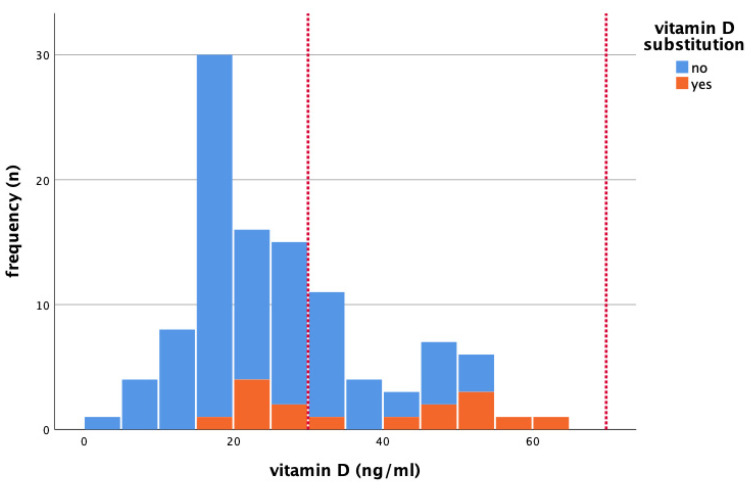
Serum 25-hydroxyvitamin D (25(OH)D) levels (ng/mL) at baseline visit in patients with and without vitamin D substitution. Reference for standard values: 30–100 ng/mL (see area in dashed red lines). (*n*) = absolute frequencies.

**Figure 2:**
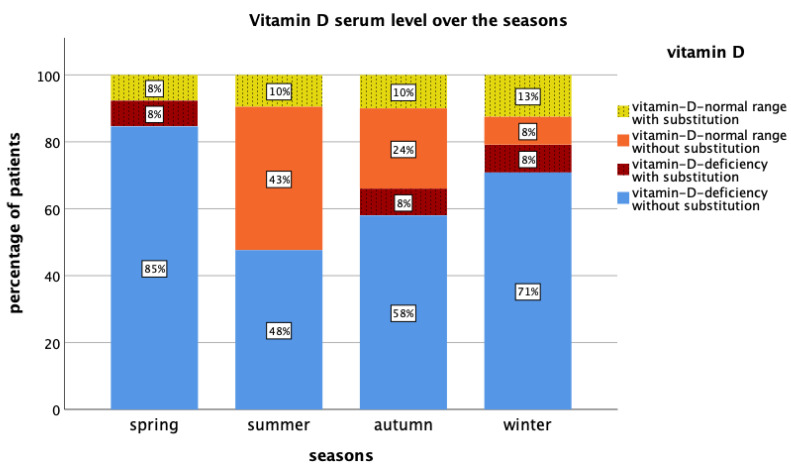
Serum 25-hydroxyvitamin D (25(OH)D) levels throughout the seasons with and without vitamin D substitution. Median vitamin D levels in spring (March, April, May) 20 ng/mL, summer (June, July, August) 31 ng/mL, autumn (September, October, November) 25 ng/mL and winter (December, January, February) 17 ng/mL. (Reference for standard vitamin D values: 30–100 ng/mL).

In all patients, median serum 25(OH)D value was 24 ng/mL (range 5–65 ng/mL) (reference for standard values: 30–100 ng/mL) [5]. A total of 18 patients (16.4%) took vitamin D supplements at the initiation visit. Median serum 25(OH)D levels were higher among the 18 patients that reported the use of vitamin D supplements (43 ng/mL versus 22 ng/mL; *p* < 0.001). The 25(OH)D was below/within/above the recommended values in 64.8%/35.2%/0% of all patients, respectively. Figure 1 shows the distribution of serum 25(OH)D levels at baseline visit. Serum 25(OH)D throughout the seasons is presented in Figure 2.

A total of 91 of the 110 (83%) patients filled in the questionnaire on sun exposure during the study. A total of 90 of the 91 answering patients (98.8%) knew that UV radiation is needed to produce vitamin D. For this reason, 41 patients (45.1%) stated that they spent more time in the sun. Nevertheless, 90 patients (98.8%) were informed that sun exposure can lead to genetic damage and the development of skin cancer. For this reason, 52 patients (57.1%) stated that they spent less time in the sun. A total of 49 patients (53.8%) classified themselves as skin type I/II (light skin color, red or blond hair, blue or green eyes). A total of 42 patients (46.2%) reported skin type III (medium skin color, dark hair, brown eyes). Daily sun exposure, use of sun protection, and avoidance of “sunny hours” are shown in Table 4, Table 5 and Table 6.

Different lifestyle and prognostic factors were examined regarding an association to serum 25(OH)D in newly diagnosed breast cancer patients (see Table 7).

Linear regression was performed to identify possible influencing lifestyle factors on 25(OH)D. Vitamin D substitution led to a significantly higher vitamin D level (regression coefficient 14, *p* < 0.001). Patients’ age, BMI, alcohol, smoking, skin type, and prior cancer history showed no significant influence on vitamin D levels. To determine seasonal effects on median 25(OH)D levels, data were split into spring (March, April, May), summer (June, July, August), autumn (September, October, November), and winter (December, January, February). Median 25(OH)D levels were 20 ng/mL (range 10–54 ng/mL) in spring, 31 ng/mL (range 11–53 ng/mL) in summer, 25 ng/mL (range 6–65 ng/mL) in autumn, and 17 ng/mL (range 5–63 ng/mL) in winter. Patients whose 25(OH)D levels were measured in summer had a vitamin D level that was seven points higher on average than in other seasons (*p* = 0.03, regression coefficient 7). Measurements of 25(OH)D levels in winter led to a six point lower vitamin D level, but without reaching statistical significance (*p* = 0.052, regression coefficient: −6). Determination of serum 25(OH)D levels in spring or autumn did not lead to statistical differences compared to other seasons (spring: *p* = 0.18, regression coefficient: −5; autumn: *p* = 0.44, regression coefficient: 2). Patients who stated that they stayed longer in the sun had higher serum 25(OH)D levels (regression coefficient: spring 1.1, summer 0.18, autumn 0.23, winter 1.2). However, none of the analyses were statistically significant (*p*-values: spring 0.32, summer 0.85, autumn 0.83, winter 0.26).

To test whether prognostic factors for breast cancer were associated with serum 25(OH)D deficiency, the patients were divided into three groups: at least mild (≤/>30 ng/mL), at least moderate (≤/>20 ng/mL), and severe (≤/>10 ng/mL) vitamin D deficiency. Results are presented in Table 8. A significant association could be seen in the group with moderate serum 25(OH)D deficiency: patients who suffered moderate vitamin D deficiency were less likely to have triple negative breast cancer (2.3% versus 14.4% of patients with serum 25(OH)D > 20 ng/mL). On the other hand, patients who suffered moderate vitamin D deficiency were more likely to have Luminal B carcinomas (32.6% versus 13.8% of patients with serum 25(OH)D > 20 ng/mL). All other prognostic factors, such as Ki67, grading, tumor subtypes (Her2 positive and Luminal A), and tumor stage (cT and cN+) did not reach significant associations with different serum 25(OH)D levels, see Table 8.

## 4. Discussion

Some studies suggest that higher vitamin D levels might reduce the risk of developing breast cancer [14,27,28,29,30]. Animal studies have also shown that vitamin D deficiency might play a role in primary tumor growth and the development of metastases in breast cancer cells [31], and an association between low vitamin D receptor expression and occurrence of more aggressive breast cancer has been seen [32]. The vitamin D receptor is commonly expressed in breast tissues, as well as in breast cancer cells [33]. It could be proven that vitamin D binds to vitamin D response elements of the promoter region of cells and thus regulates vitamin D-dependent genes [7]. A possible explanation of how vitamin D might affect breast cancer biology might be the tumor suppressive effects of vitamin D receptor catabolism, as a defect in the vitamin D receptor (e.g., CYP27B1 and CYP24A1 regulation) led to decreased signaling [33]. In addition, it could be shown that non-coding RNAs (lncRNAs) play an important role in the pathogenesis of breast cancer [34]. By modulating IncRNA, vitamin D could have a protective effect on the development of breast cancer [34]. However, by now these results could mostly be shown in vitro and in animal experiments, not in clinical trials [7,8,9]. Thus, a general benefit of vitamin D substitution in cancer patients or influence on therapy success and outcome could not yet been proven [1,35].

In the present study, we showed that almost two thirds of newly diagnosed German breast cancer patients suffer from vitamin D deficiency. The median vitamin D serum concentration of all patients within this study was below the recommended minimum value (median vitamin D level of 24 ng/mL; vitamin D reference for standard values: 30–100 ng/mL) [5]. As expected, patients who consumed supplements had higher vitamin D levels on average. However, despite supplementation, seven out of 17 patients still showed mild vitamin D deficiency. None of the patients were above recommended reference values. This supports the hypothesis that vitamin D intoxication, and subsequent hypercalcemia and hyperphosphatemia, are extremely rare [5]. A Canadian study also showed similarly high rates of vitamin D deficiency in breast cancer patients [36]. Vitamin D deficiency seemed higher in the present study (64.8%) compared to the healthy, cancer free German population, where in the year 1991 57.8% of women and 56.8% of men suffered from vitamin D deficiency [4]. Nevertheless, lifestyle and nutrition has changed within the last 30 years, which could also be a possible explanation for this difference. A recent systematic review indicated the importance of exercise interventions as well as supplement interventions (e.g., vitamin D) in breast cancer patients, and their positive effects on cardiorespiratory fitness, body composition, and quality of life [37].

Another systematic review and meta-analysis of 22 observational studies with a cumulative sample size of 229,597 subjects associated low serum 25(OH)D levels with breast cancer occurrence [38]. Despite this, a definite connection and pathogenesis between breast cancer development and vitamin D levels has not yet been proven [3]. Nevertheless, breast cancer patients might be particularly susceptible to suffering complications of vitamin D deficiency, such as bone fractures, due to additional risk factors caused by the disease itself. It is known that vitamin D deficiency is associated with long-term mortality especially in hospitalized, malnourished patients [39]. In breast cancer patients, disease associated immobilization, chemotherapy, endocrine therapy, radiotherapy, and metastases may further increase bone loss and the risk of fractures. For example, hormone receptor positive breast cancer patients who are treated with endocrine therapy (e.g., aromatase inhibitors) suffer from bone loss and osteoporosis due to reduced estrogen levels [40]. In addition, vitamin D deficiency can lead to negative side effects during oncological treatment, such as higher risk of polyneuropathy under paclitaxel chemotherapy [41]. Thus, in breast cancer patients, special attention should be paid to achieve and maintain vitamin D levels within the reference range [3].

We observed higher vitamin D levels in summer compared to other seasons. Furthermore, patients who stated that they stayed longer in the sun had higher vitamin D levels. Even if this did not reach statistical significance due to the limited number of patients, our observations agree with previous population-based publications that describe seasonal variations of vitamin D levels due to varying sunlight exposure [42,43,44]. Sufficient vitamin D values at the end of summer do not prevent vitamin D deficiency in winter [44]. This illustrates the need for vitamin D monitoring and supplementation in clinical routine, especially in highly vulnerable cancer patients [1].

Recent meta-analyses showed that vitamin D was associated with breast cancer prognosis and breast cancer mortality [45]. Particularly aggressive node positive and triple negative (ER negative, PR negative, Her2 negative) carcinomas were linked to reduced vitamin D levels [13,14,46]. Furthermore, breast cancer patients with circulating tumor cells in peripheral blood (who were therefore at risk of more invasive disease) were associated with lower vitamin D levels [47]. In a systematic review with 13,135 breast cancer patients, low vitamin D levels were especially associated with triple negative breast cancer [48]. Furthermore, in a prospective cohort study in California, 3175 women suffering from incident breast cancer were enrolled within two months of diagnoses and 25(OH)D was determined: 25(OH)D was lower in patients with advanced tumor stages and aggressive tumors (e.g., triple negative breast cancer) [49]. They even saw an association between 25(OH)D and invasive-disease-free survival, as well as overall survival [49]. This contrasts with the present study. Patients who suffered moderate vitamin D deficiency were less likely to have triple negative breast cancer and we saw no association between 25(OH)D and other prognostic factors (e.g., tumor stage, grading, Ki67). Nevertheless, patients with moderate 25(OH)D deficiency had a higher rate of Luminal B tumors (which are more aggressive than Luminal A tumors). A possible explanation for the different results in our study compared to current literature might be the time of blood sampling and 25(OH)D level determination. In the present study, all patients received blood sampling at the time of diagnosis and prior to the start of treatment. In particular, patients with aggressive (e.g., triple negative carcinomas) or advanced tumor stages (cT3/4, cN+) were treated with (neo-) adjuvant chemotherapy, and it is well known that chemotherapy can lead to decreased 25(OH)D levels [50,51,52]. In the cited studies [48,49], the time of 25(OH)D level determination was very heterogenous. Further studies with 25(OH)D level determination before the start of therapy are needed to investigate the true association of prognostic factors and 25(OH)D level.

Limitations of the study are the limited number of patients, and the fact that supplementation habits and sun exposure were retrospectively reported by the patients themselves. Nevertheless, these results give important insights into the serum 25(OH)D levels of newly diagnosed breast cancer patients, which have rarely been studied.

## 5. Conclusions

Almost two thirds of all breast cancer patients within this study suffered from vitamin D deficiency. Efforts should be made to include 25(OH)D level determination in clinical routine and to substitute vitamin D deficient patients to prevent side effects and long-term consequences of vitamin D deficiency. In the current study, patients suffering moderate vitamin D deficiency were less likely to have triple negative breast cancer but were more likely to have Luminal B carcinomas. Associations between 25(OH)D level and other prognostic factors in breast cancer (e.g., tumor stage, subtype, Ki67, grading) could not be proven. Further studies are needed to explore the effects of vitamin D deficiency in breast cancer patients during oncological treatment.

## Figures and Tables

**Table 1:** Tumor biology and serum 25-hydroxyvitamin D (25(OH)D) levels. “ER” = estrogen receptor, “PR” = progesterone receptor.

Tumor Biology	Frequency (*n*)	Percentage (%)	25(OH)D (Median (Min–Max))
Luminal A (ER and/or PR positive, Ki67 < 25)	48	43.6	23 ng/mL (5–65 ng/mL)
Luminal B (ER and/or PR positive, Ki67 ≥ 25)	24	21.9	19 ng/mL (10–63 ng/mL)
Her2 positive (ER/PR positive or negative)	27	24.5	24 ng/mL (12–57 ng/mL)
Triple negative (ER, PR and Her2 negative)	11	10.0	30 ng/mL (20–54 ng/mL)
Total	110	100	24 ng/mL (5–65 ng/mL)

**Table 2:** Tumor entity and serum 25-hydroxyvitamin D (25(OH)D) levels. “NST” = No special type.

Tumor Entity	Frequency (*n*)	Percentage (%)	25(OH)D (Median (Min–Max))
NST	91	82.7	24 ng/mL (5–65 ng/mL)
Invasive lobular	12	10.9	29 ng/mL (7–53 ng/mL)
Inflammatory	2	1.8	32 ng/mL (11–53 ng/mL)
Mucinous	1	0.9	15 ng/mL
Tubular	2	1.8	16 ng/mL
Metaplastic	1	0.9	12 ng/mL
Mixed (NST, tubular)	1	0.9	47 ng/mL
Total	110	100	24 ng/mL (5–65 ng/mL)

**Table 3:** Tumor stage and serum 25-hydroxyvitamin D (25(OH)D) levels.

cT	Frequency (*n*)	Percentage (%)	25(OH)D (Median (Min–Max))
cT0 *	3	2.7	31 ng/mL (27–57 ng/mL)
cT1	71	64.5	22 ng/mL (5–65 ng/mL)
cT2	31	28.2	24 ng/mL (6–48 ng/mL)
cT3	1	0.9	30 ng/mL
cT4	4	3.6	38 ng/mL (11–53 ng/mL)
cN	Frequency (*n*)	Percentage (%)	
cN0	83	75.5	25 ng/mL (5–65 ng/mL)
cN+	27	24.5	23 ng/mL (6–57 ng/mL)
M	Frequency (*n*)	Percentage (%)	
M0	110	100	24 ng/mL (5–65 ng/mL)
Grading	Frequency (*n*)	Percentage (%)	
G1	10	9.2	30 ng/mL (13–47 ng/mL)
G2	56	51.4	22 ng/mL (5–65 ng/mL)
G3	43	39.4	26 ng/mL (10–63 ng/mL)
Total	110	100	24 ng/mL (5–65 ng/mL)

* 3 patients had a recurrent tumor in the lymph nodes without tumor manifestation in the breast, thus cT0.

**Table 4:** Daily sun exposure. The specified values in the table refer to the recommendations of the German Society of Nutrition e.V. (https://www.dge.de/wissenschaft/faqs/vitamin-d/, accessed on 14 March 2023).

**Spring**	**Frequency (*n*)**	**Percentage (%)**
<10 min	25	27.5
10–20 min	26	28.6
15–25 min	12	13.2
25–50 min	19	20.9
>50 min	9	9.9
**Summer**	**Frequency (*n*)**	**Percentage (%)**
<5 min	16	17.6
5–10 min	17	18.7
10–15 min	15	16.5
15–30 min	17	18.7
>30 min	26	28.6
**Autumn**	**Frequency (*n*)**	**Percentage (%)**
<10 min	21	23.1
10–20 min	22	24.2
15–25 min	12	13.2
25–50 min	25	27.5
>50 min	11	12.1
**Winter**	**Frequency (*n*)**	**Percentage (%)**
<10 min	35	38.5
10–20 min	17	18.7
15–25 min	15	16.5
25–50 min	16	17.6
>50 min	8	8.8

**Table 5:** Use of sun protection.

**Spring**	**Frequency (*n*)**	**Percentage (%)**
Never	50	54.9
1–3 days/week	21	23.1
3–6 days/week	6	6.6
Every day	14	15.4
**Summer**	**Frequency (*n*)**	**Percentage (%)**
Never	6	6.6
1–3 days/week	32	35.2
3–6 days/week	22	24.2
Every day	31	34.1
**Autumn**	**Frequency (*n*)**	**Percentage (%)**
Never	49	53.8
1–3 days/week	25	27.5
3–6 days/week	8	8.8
Every day	9	9.9
**Winter**	**Frequency (*n*)**	**Percentage (%)**
Never	69	75.8
1–3 days/week	11	12.1
3–6 days/week	4	4.4
Every day	7	7.7

**Table 6:** Avoidance of going outdoors during “sunny hours” (from 12:00 p.m. to 4:00 p.m.).

**Spring**	**Frequency (*n*)**	**Percentage (%)**
Never	52	57.1
1–3 days/week	11	12.1
3–6 days/week	6	6.6
Every day	22	24.2
**Summer**	**Frequency (*n*)**	**Percentage (%)**
Never	15	16.5
1–3 days/week	23	25.3
3–6 days/week	19	20.9
Every day	34	37.4
**Autumn**	**Frequency (*n*)**	**Percentage (%)**
Never	52	57.1
1–3 days/week	15	16.5
3–6 days/week	4	4.4
Every day	20	22.0
**Winter**	**Frequency (*n*)**	**Percentage (%)**
Never	62	68.1
1–3 days/week	7	7.7
3–6 days/week	6	6.6
Every day	16	17.6

**Table 7:** Different lifestyle and prognostic factors were examined regarding an association to serum 25-hydroxyvitamin D (25(OH)D).

**Lifestyle factors**	Age, BMI, alcohol, smoking, skin type, prior cancer history, season of blood sampling (spring, summer, autumn, winter), daily sun exposure (spring, summer, autumn, winter), 25(OH)D substitution
**Prognostic factors**	Ki67, grading, triple negative carcinomas, Her2 positive carcinomas, Luminal A carcinomas, Luminal B carcinomas, cT (tumor stage), cN (tumor stage)

**Table 8:** Association of prognostic factors for breast cancer with serum 25-hydroxyvitamin D (25(OH)D) deficiency. The patients were divided into three groups: severe (≤10 µg/mL: five patients versus >10 µg/L: 103 patients), moderate (≤20 µg/mL: 43 patients versus >20 µg/L: 65 patients), and mild (≤30 µg/mL: 74 patients versus >30 µg/L: 34 patients) vitamin D deficiency.

25(OH)D Levels	≤ vs. >10 µg/L	≤ vs. >20 µg/L	≤ vs. >30 µg/L
Ki67	*p* = 0.33	*p* = 0.07	*p* = 0.39
Grading	*p* = 0.10	*p* = 0.61	*p* = 0.74
Triple negative	*p* = 1.00	*p* = 0.047	*p* = 0.74
Her2 positive	*p* = 0.33	*p* = 0.50	*p* = 1.00
Luminal A	*p* = 0.65	*p* = 1.00	*p* = 0.68
Luminal B	*p* = 0.29	*p* = 0.03	*p* = 0.62
cT (tumor stage)	*p* = 0.95	*p* = 0.50	*p* = 0.45
cN+ (lymph nodes)	*p* = 0.60	*p* = 1.00	*p* = 0.34

Mann–Whitney U test was used for metric and ordinal variables with asymptotic *p*-value. For the group with severe vitamin D deficiency, exact *p*-value was used due to small sample size. Nominal variables were tested with crosstabulation and Chi-Square test as well as Fishers’ exact *p*-value.

## Data Availability

The datasets generated during the current study are available from the corresponding author on reasonable request.

## References

[1] Gröber U., Holzhauer P., Kisters K., Holick M.F., Adamietz I.A. (2016). Micronutrients in oncological intervention. Nutrients.

[2] Gröber U., Hübner J., Holzhauer P., Kleeberg U.R. (2010). Antioxidanzien und andere Mikronährstoffe in der komplementären Onkologie. Onkologe.

[3] Amrein K., Scherkl M., Hoffmann M., Neuwersch-Sommeregger S., Köstenberger M., Tmava Berisha A., Martucci G., Pilz S., Malle O. (2020). Vitamin D deficiency 2.0: An update on the current status worldwide. Eur. J. Clin. Nutr..

[4] Hintzpeter B., Mensink G.B.M., Thierfelder W., Müller M.J., Scheidt-Nave C. (2008). Vitamin D status and health correlates among German adults. Eur. J. Clin. Nutr..

[5] Holick M.F. (2007). Vitamin D Deficiency. N. Engl. J. Med..

[6] Galesanu C., Mocanu V. (2015). Vitamin D deficiency and the clinical consequences. Rev. Med. Chir. Soc. Med. Nat. Iasi.

[7] Linowiecka K., Wolnicka-Głubisz A., Brozyna A. (2021). Vitamin D endocrine system in breast cancer. Acta Biochim. Pol..

[8] De La Puente-Yagüe M., Cuadrado-Cenzual M.A., Ciudad-Cabañas M.J., Hernández-Cabria M., Collado-Yurrita L. (2018). Vitamin D: And its role in breast cancer. Kaohsiung J. Med. Sci..

[9] Shao T., Klein P., Grossbard M.L. (2012). Vitamin D and Breast Cancer. Oncologist.

[10] Vanhevel J., Verlinden L., Doms S., Wildiers H., Verstuyf A. (2022). The role of vitamin D in breast cancer risk and progression. Endocr. Relat. Cancer.

[11] Filip-Psurska B., Zachary H., Strzykalska A., Wietrzyk J. (2022). Vitamin D, Th17 Lymphocytes, and Breast Cancer. Cancers.

[12] Segovia-Mendoza M., García-Quiroz J., Díaz L., García-Becerra R. (2021). Combinations of Calcitriol with Anticancer Treatments for Breast Cancer: An Update. Int. J. Mol. Sci..

[13] Blasiak J., Pawlowska E., Chojnacki J., Szczepanska J., Fila M., Chojnacki C. (2020). Vitamin D in triple-negative and BRCA1-deficient breast cancer—Implications for pathogenesis and therapy. Int. J. Mol. Sci..

[14] Lope V., Castelló A., Mena-Bravo A., Amiano P., Aragonés N., Fernández-Villa T., Guevara M., Dierssen-Sotos T., Fernandez-Tardon G., Castano-Vinyals G. (2018). Serum 25-hydroxyvitamin D and breast cancer risk by pathological subtype (MCC-Spain). J. Steroid Biochem. Mol. Biol..

[15] Min K.-W., Kim D.-H., Do S.-I., Pyo J.-S., Chae S.W., Sohn J.H., Kim K., Lee H.J., Kim D.H., Oh S. (2016). High Ki67/BCL2 index is associated with worse outcome in early stage breast cancer. Postgrad Med. J..

[16] Yang Z.J., Yu Y., Chi J.R., Guan M., Zhao Y., Cao X.C. (2018). The combined pN stage and breast cancer subtypes in breast cancer: A better discriminator of outcome can be used to refine the 8th AJCC staging manual. Breast Cancer.

[17] Bardia A., Hurvitz S. (2018). Targeted Therapy for Premenopausal Women with HR+, HER2− Advanced Breast Cancer: Focus on Special Considerations and Latest Advances. Clin. Cancer Res..

[18] Giuliano A.E., Connolly J.L., Edge S.B., Mittendorf E.A., Rugo H.S., Solin L.J., Weaver D.L., Winchester D.J., Hortobagyi G.N. (2017). Breast Cancer-Major changes in the American Joint Committee on Cancer eighth edition cancer staging manual. CA Cancer J. Clin..

[19] Osmani F., Hajizadeh E., Rasekhi A., Akbari M.E. (2019). Prognostic factors associated with locoronal relapses, metastatic relapses, and death among women with breast cancer. Population-based cohort study. Breast.

[20] Tan Q.-X., Qin Q.-H., Yang W.-P., Mo Q.-G., Wei C.-Y. (2014). Prognostic value of Ki67 expression in HR-negative breast cancer before and after neoadjuvant chemotherapy. Int. J. Clin. Exp. Pathol..

[21] (2021). Interdisziplinäre S3-Leitlinie für Die Früherkennung, Diagnostik, Therapie und Nachsorge des Mammakarzinoms.

[22] Li X., Yang J., Peng L., Sahin A.A., Huo L., Ward K.C., O’Regan R., Torres M.A., Meisel J.L. (2017). Triple-negative breast cancer has worse overall survival and cause-specific survival than non-triple-negative breast cancer. Breast Cancer Res. Treat..

[23] Zemlin C., Stuhlert C., Schleicher J.T., Wörmann C., Altmayer L., Lang M., Scherer L.-S., Thul I.C., Muller C., Kaiser E. (2021). Longitudinal assessment of physical activity, fitness, body composition, immunological biomarkers, and psychological parameters during the first year after diagnosis in women with non-metastatic breast cancer: The BEGYN study protocol. Front. Oncol..

[24] James D., Gospodarowicz M.K., Wittekind C. (2016). TNM Classification of Malignant Tumours.

[25] Elston C.W., Ellis I.O. (1991). Pathological prognostic factors in breast cancer, I. The value of histological grade in breast cancer: Experience from a large study with long-term follow-up. Histopathology.

[26] Nielsen T.O., Leung S.C.Y., Rimm D.L., Dodson A., Acs B., Badve S., Denkert C., Ellis M.J., Fineberg S., Flowers M. (2021). Assessment of Ki67 in Breast Cancer: Updated Recommendations from the International Ki67 in Breast Cancer Working Group. JNCI J. Natl. Cancer Inst..

[27] Welsh J.E. (2021). Vitamin D and breast cancer: Mechanistic update. JBMR Plus.

[28] Giovannucci E. (2009). Vitamin D and cancer incidence in the Harvard Cohorts. Ann. Epidemiol..

[29] Shamsi U., Khan S., Azam I., Khan A.H., Maqbool A., Hanif M., Gill T., Iqbal R., Callen D. (2020). A multicenter case control study of association of vitamin D with breast cancer among women in Karachi, Pakistan. PLoS ONE.

[30] Estébanez N., Gómez-Acebo I., Palazuelos C., Llorca J., Dierssen-Sotos T. (2018). Vitamin D exposure and Risk of Breast Cancer: A meta-analysis. Sci. Rep..

[31] Williams J.D., Aggarwal A., Swami S., Krishnan A.V., Ji L., Albertelli M.A., Feldman B.J. (2016). Tumor autonomous effects of vitamin D deficiency promote breast cancer metastasis. Endocrinology.

[32] Al-Azhri J., Zhang Y., Bshara W., Zirpoli G., McCann S.E., Khoury T., Morisson C.D., Edge S.B., Ambrosone C.B., Yao S. (2017). Tumor Expression of Vitamin D Receptor and Breast Cancer Histopathological Characteristics and Prognosis. Clin. Cancer Res..

[33] Voutsadakis I.A. (2020). Vitamin D receptor (VDR) and metabolizing enzymes CYP27B1 and CYP24A1 in breast cancer. Mol. Biol. Rep..

[34] Blasiak J., Chojnacki J., Pawlowska E., Jablkowska A., Chojnacki C. (2022). Vitamin D May Protect against Breast Cancer through the Regulation of Long Noncoding RNAs by VDR Signaling. Int. J. Mol. Sci..

[35] Harvie M. (2014). Nutritional supplements and cancer: Potential benefits and proven harms. Am. Soc. Clin. Oncol. Educ. Book.

[36] Rosso C., Fera N., Murugan N.J., Voutsadakis I.A. (2022). Vitamin D Levels in Newly Diagnosed Breast Cancer Patients according to Tumor Sub-Types. J. Diet Suppl..

[37] Pérez-Bilbao T., Alonso-Dueñas M., Peinado A.B., San Juan A.F. (2023). Effects of Combined Interventions of Exercise and Diet or Exercise and Supplementation on Breast Cancer Patients: A Systematic Review. Nutrients.

[38] Hossain S., Beydoun M.A., Beydoun H.A., Chen X., Zonderman A.B., Wood R.J. (2019). Vitamin D and breast cancer: A systematic review and meta-analysis of observational studies. Clin. Nutr. ESPEN.

[39] Merker M., Amsler A., Pereira R., Bolliger R., Tribolet P., Braun N., Hoess C., Pavlicek V., Bilz S., Sigrist S. (2019). Vitamin D deficiency is highly prevalent in malnourished inpatients and associated with higher mortality. Medicine.

[40] Gaudio A., Xourafa A., Rapisarda R., Castellino P. (2022). Therapeutic Options for the Management of Aromatase Inhibitor- Associated Bone Loss. Endocr. Metab. Immune Disord. Drug Targets.

[41] Jennaro T.S., Fang F., Kidwell K.M., Smith E.M.L., Vangipuram K., Burness M.L., Griggs J.J., Van Poznak C., Hayes D.F., Henry N.L. (2020). Vitamin D deficiency increases severity of paclitaxel-induced peripheral neuropathy. Breast Cancer Res. Treat..

[42] Poskitt E.M.E., Cole T.J., Lawson D.E.M. (1979). Diet, sunlight, and 25-hydroxy vitamin D in healthy children and adults. Br. Med. J..

[43] Heidari B., Mirghassemi M.B.H. (2012). Seasonal variations in serum vitamin D according to age and sex. Caspian J. Intern. Med..

[44] Shakeri H., Pournaghi S.J., Hashemi J., Mohammad-Zadeh M., Akaberi A. (2017). Do sufficient vitamin D levels at the end of summer in children and adolescents provide an assurance of vitamin D sufficiency at the end of winter? A cohort study. J. Pediatr. Endocrinol. Metab..

[45] Park S.-H., Hoang T., Kim J. (2021). Dietary Factors and Breast Cancer Prognosis among Breast Cancer Survivors: A Systematic Review and Meta-Analysis of Cohort Studies. Cancers.

[46] Buono G., Giuliano M., De Angelis C., Lauria R., Forestieri V., Pensabene M., Bruzzese D., De Placido S., Arpino G. (2017). Pretreatment Serum Concentration of Vitamin D and Breast Cancer Characteristics: A Prospective Observational Mediterranean Study. Clin. Breast Cancer.

[47] Mego M., Vlkova B., Minarik G., Cierna Z., Karaba M., Benca J., Sedlackova T., Cholujova D., Gronesova P., Kalavska K. (2022). Vitamin D and circulating tumor cells in primary breast cancer. Front. Oncol..

[48] Tommie J.L., Pinney S.M., Nommsen-Rivers L.A. (2018). Serum Vitamin D Status and Breast Cancer Risk by Receptor Status: A Systematic Review. Nutr. Cancer.

[49] Yao S., Kwan M.L., Ergas I.J., Roh J.M., Cheng T.-Y.D., Hong C.-C., McCann S.E., Tang L., Davis W., Liu S. (2017). Association of Serum Level of Vitamin D at Diagnosis With Breast Cancer Survival. JAMA Oncol..

[50] Kim H.J., Koh B.S., Yu J.H., Lee J.W., Son B.H., Kim S.B., Ahn S.H. (2014). Changes in serum hydroxyvitamin D levels of breast cancer patients during tamoxifen treatment or chemotherapy in premenopausal breast cancer patients. Eur. J. Cancer.

[51] Kok D.E., van den Berg M.M.G.A., Posthuma L., van ’t Erve I., van Duijnhoven F.J.B., de Roos W.K., Grosfeld S., Los M., Sommeijer D.W., van Laarhoven H.W.M. (2019). Changes in Circulating Levels of 25-hydroxyvitamin D3 in Breast Cancer Patients Receiving Chemotherapy. Nutr. Cancer.

[52] Kim J.S., Haule C.C., Kim J.H., Lim S.M., Yoon K.H., Kim J.Y., Park H.S., Park S., Kim S.I., Cho Y.U. (2018). Association between Changes in Serum 25-Hydroxyvitamin D Levels and Survival in Patients with Breast Cancer Receiving Neoadjuvant Chemotherapy. J. Breast Cancer.

